# Implementable Prediction of Pressure Injuries in Hospitalized Adults: Model Development and Validation

**DOI:** 10.2196/51842

**Published:** 2024-05-08

**Authors:** Thomas J Reese, Henry J Domenico, Antonio Hernandez, Daniel W Byrne, Ryan P Moore, Jessica B Williams, Brian J Douthit, Elise Russo, Allison B McCoy, Catherine H Ivory, Bryan D Steitz, Adam Wright

**Affiliations:** ^1^Department of Biomedical Informatics, Vanderbilt University Medical Center, Nashville, TN, United States; 2 Department of Biostatistics, Vanderbilt University Medical Center, Nashville, TN, United States; 3 Department of Anesthesiology, Division of Critical Care Medicine, Vanderbilt University Medical Center, Nashville, TN, United States; 4 Department of Nursing, Vanderbilt University Medical Center, Nashville, TN, United States

**Keywords:** patient safety, electronic health record, EHR, implementation, predictive analytics, prediction, injury, pressure injury, hospitalization, adult, development, routine care, prediction model, pressure sore

## Abstract

**Background:**

Numerous pressure injury prediction models have been developed using electronic health record data, yet hospital-acquired pressure injuries (HAPIs) are increasing, which demonstrates the critical challenge of implementing these models in routine care.

**Objective:**

To help bridge the gap between development and implementation, we sought to create a model that was feasible, broadly applicable, dynamic, actionable, and rigorously validated and then compare its performance to usual care (ie, the Braden scale).

**Methods:**

We extracted electronic health record data from 197,991 adult hospital admissions with 51 candidate features. For risk prediction and feature selection, we used logistic regression with a least absolute shrinkage and selection operator (LASSO) approach. To compare the model with usual care, we used the area under the receiver operating curve (AUC), Brier score, slope, intercept, and integrated calibration index. The model was validated using a temporally staggered cohort.

**Results:**

A total of 5458 HAPIs were identified between January 2018 and July 2022. We determined 22 features were necessary to achieve a parsimonious and highly accurate model. The top 5 features included tracheostomy, edema, central line, first albumin measure, and age. Our model achieved higher discrimination than the Braden scale (AUC 0.897, 95% CI 0.893-0.901 vs AUC 0.798, 95% CI 0.791-0.803).

**Conclusions:**

We developed and validated an accurate prediction model for HAPIs that surpassed the standard-of-care risk assessment and fulfilled necessary elements for implementation. Future work includes a pragmatic randomized trial to assess whether our model improves patient outcomes.

## Introduction

Pressure injuries comprise damage to skin and underlying tissue that usually occurs over a bony prominence but can be related to placement of medical devices [1]. The injury occurs because of intense or prolonged pressure that is combined with shear forces. Pressure injuries are a widespread and costly problem. A recent study found the prevalence of pressure injuries may be close to 30% for patients in intensive care units, which is 10% higher than previous estimates [2,3]. Patients with pressure injuries experience pain and the potential for infection and debilitation, which prolongs hospital stays and impacts recovery. Furthermore, increasing evidence supports the association between severity of pressure injuries and patient mortality [2]. In the United States, health care systems absorb on average US $10,000 per hospital-acquired pressure injury (HAPI), which contributes to a cost burden that will soon exceed US $30 billion [4,5].

Prevention of pressure injuries requires an accurate risk assessment and an interdisciplinary approach with routine repositioning, maintaining dry skin, and padding pressure points to reduce injury [6-8]. Currently, health care systems are striving to accurately measure and prevent HAPIs, since they can be common and negatively impact patient care [9]. Patient factors such as age, vasopressor support, mechanical ventilation, low albumin, and renal failure can increase the risk for pressure injuries [10,11]. Multiple standardized risk assessment tools have been developed to systematically assess patient factors and assist clinicians in identifying at-risk patients [12,13]. Of these tools, the Braden scale has remained the standard of care across health systems for decades. The Braden scale incorporates components of sensory perception, activity, mobility, and nutrition, as well as skin moisture, friction, and shear force, to produce a score that indicates the risk of developing a pressure injury [14]. Although use of the Braden scale is widespread, its accuracy and reliability in diverse settings and patients is in question; thus, researchers have turned to more advanced risk prediction models that incorporate additional patient factors [12,13,15,16].

**Figure 1.**
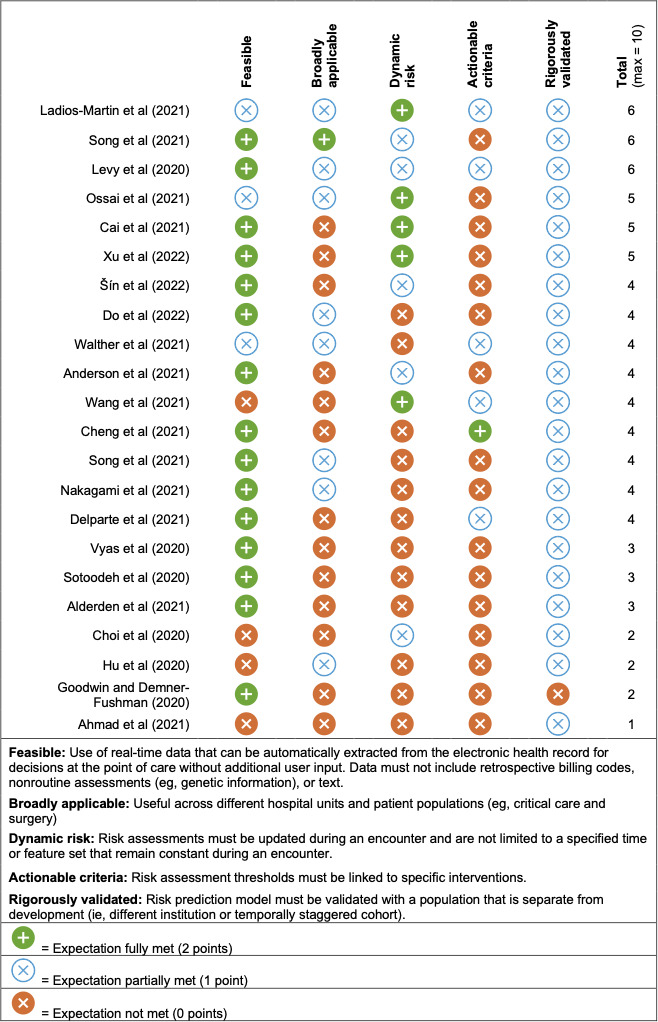
Comparison of current pressure injury prediction models according to elements of implementable models [25-45].

Recent literature reviews of advanced risk prediction models have highlighted excellent performance in predicting pressure injuries [17-21]. Zhou and colleagues [20] found that 74% of studies achieved an area under the receiver operating curve (AUC) between 0.68 and 0.99. Although these models were exceptionally accurate at predicting pressure injuries, no studies to our knowledge have implemented such models to reduce the number of pressure injuries. Numerous prediction models have been developed across clinical domains, but few have improved patient outcomes, leading researchers to identify a variety of required elements that may be necessary to implement prediction models in practice [22-24]. For instance, Randall Moorman [23] proposed properties, such as change of risk over time (eg, dynamic risk), for predictive analytics in neonatal intensive care units. Keim-Malpass and colleagues [24] found that potential users want prediction tools to be integrated with the electronic health record (EHR; eg, feasibility). We reviewed and agreed upon 5 elements that applied to HAPI prediction (ie, it should be feasible, broadly applicable, include dynamic risk and actionable criteria, and be rigorously validated) and then applied these elements to 22 recent models from 2020 to 2022 (Figure 1) [17,20,21]. We found no models fulfilled all the necessary elements to impact patient care. To help bridge the gap from model development to implementation, the objective of this study was, therefore, to develop and validate a model that fulfilled these elements and then compare its performance to usual care (ie, the Braden scale).

## Methods

### Study Population

We used retrospective data from the EHR at Vanderbilt University Medical Center between January 1, 2018, and July 1, 2022. All hospital admissions were included if the length of stay was longer than 24 hours and patient age was greater than 18 years on admission. HAPIs were identified using nurse flowsheet documentation. Nurses use flowsheets to document a variety of assessments, with our institution using a dedicated section for pressure injuries. The presence or absence of a pressure injury is assessed on admission and daily for each patient in the hospital. If a pressure injury is identified, the nurse documents whether it was present on admission and additional characteristics of the pressure injury, including the stage and location. We considered pressure injuries documented with a “no” in the column “present on admission” as HAPIs. For patients who had more than one HAPI, we used the first documented. The cohort included 197,911 hospitalizations, 129,100 patients, and 5458 HAPIs.

### Feature Selection and Cohort Development

**Figure 2.**
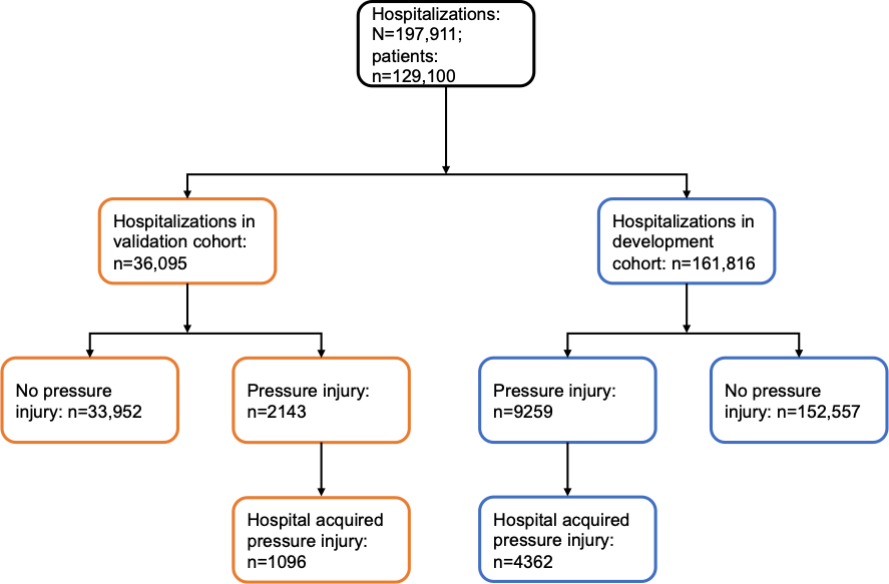
Model development and validation cohorts.

We first identified relevant features associated with pressure injuries from the literature. The list of relevant features was supplemented and pruned by clinical domain experts and informaticians at Vanderbilt University Medical Center. In total, 51 features were extracted as candidate features for predicting HAPIs. Importantly, features were only extracted if they were available at the time of hospitalization and could be used to update the risk prediction during the encounter (ie, no claims data were used). Table 1 provides a summary of the extracted features. Missing values were imputed with the cohort median [46,47]. Multimedia Appendix 1 provides the full cohort characteristics, including missing values and a full list of measures. We split the full cohort temporally into model development and validation cohorts based on the number of events, with the development and validation cohorts including 80% and 20% of HAPIs, respectively. The development cohort included 161,816 hospitalizations and 4362 HAPIs from January 1, 2018, to August 26, 2021, and the validation cohort included 36,095 hospitalizations and 1096 HAPIs from August 27, 2021, to June 29, 2022 (Figure 2). Outcomes and features were identified and extracted in the same manner for the development and validation cohorts.

**Table 1.** Overview of extracted features.

Source	Feature
Patient demographics and social history	Age; gender; race; ethnicity; smoking status
Administration	Hospital admission through emergency department; intensive care unit admission; length of stay
Flowsheets	Hospital-acquired pressure injury (primary outcome); temperature; respiratory rate; heart rate; BMI; oxygen saturation; blood pressure; Braden scale (items and composite score); consciousness; gait transfer; Glasgow Coma Scale; malnutrition score; spinal cord injury; dialysis during hospitalization; tracheostomy; gastric tube; central line; chest tube; ostomy; drain; extracorporeal membrane oxygenation
Laboratory results	Hemoglobin; hemoglobin A_1C_; hematocrit; mean corpuscular hemoglobin concentration; red cell distribution width; platelet count; chloride; blood urea nitrogen; creatinine; lactate; albumin; glucose

### Model Development

We developed 3 models for comparison using logistic regression. The present model (Vanderbilt) used a broad set of candidate features (Table 1). The second model used the sum of the individual item measures from the Braden scale (ie, continuous Braden) [14]. Finally, since the Braden scale is typically operationalized using a single composite score (ie, less than 18=high risk; greater than or equal to 18=low risk), we included the dichotomous Braden for comparison as well. Logistic regression is the most frequently used model in clinical care [20,48]. The primary advantages of using logistic regression are that feature importance is easily interpretable and that the mathematical equation used to extract features and calculate a risk prediction is readily available in most commercial EHRs. Currently, the output from many machine learning models is not operationalizable for patient care in the EHR. To account for nonlinearity of the numeric features, we tested 3 knot-restricted cubic splines but found the discrimination failed to improve by using the nonlinear model [49]. Since the purpose was to develop a model that could be easily implemented in the EHR and compare it to standard care, we focused on use of logistic regression for the Vanderbilt and continuous Braden models.

We first included all 51 candidate features in the present (Vanderbilt) model to examine complexity versus accuracy as measured by cross-validation AUC. Again, included features were derived from the literature and refined by clinical domain experts and informaticians. We tested for multicollinearity by examining the proportion of variance in each candidate feature that could be explained by other candidate features and removed hemoglobin. Included features had to be structured and readily available for automated processing in the EHR without additional input by the user. Using the conservative 15:1 rule, we were able to include 290.8 degrees of freedom in the model. To ensure the model was broadly applicable across settings and patients, we used a least absolute shrinkage and selection operator (LASSO) approach to identify important candidate features. Candidate features were standardized (scaled and centered) prior to running the LASSO regression. LASSO introduces a penalty term to the standard regression model, which forces some of the regression coefficients to shrink toward zero, effectively performing feature selection [50]. Variables with nonzero coefficients were included in the final model. The model was designed to calculate a risk prediction on admission and daily while the patient was in the hospital. Missing numeric measures were to be imputed with the cohort median until measures became available.

### Model Evaluation

The final model was assessed in an external cohort that was temporally separated from the model development cohort. We evaluated the model using traditional and novel performance measures, which included the AUC, Brier score, slope, intercept, integrated calibration index, and calibration curve. AUC is a performance measure for the discrimination of HAPI versus no HAPI. It combines the true and false positive rates, with an AUC of 0.5 indicating no meaningful discrimination. The Brier score accounts for the predicted HAPI outcome as well as the estimate and is calculated by the squared difference between the prediction (0 to 1) and outcome (0=no HAPI and 1=HAPI) [51]. For example, if a patient had a 90% probability of developing a HAPI and did develop a HAPI during that encounter, the Brier score would be 0.01. A Brier score of 0 indicates perfect accuracy and a score of 1 indicates perfect inaccuracy. The integrated calibration index is a numeric summary of model calibration across the predicted probabilities [52]. It is the weighted average of the absolute difference between the observed and predicted probabilities; therefore, a lower integrated calibration index indicates better calibration. A slope equal to 1 indicates agreement between the observed response and the predicted probability, while a slope greater than 1 indicates potential underfitting, and a slope lower than 1 indicates potential overfitting [52]. Similarly, an intercept of zero is ideal. As with prior models, no adjustments were made for multiple comparisons [47,53,54]. We used the TRIPOD (Transparent Reporting of a Multivariable Prediction Model for Individual Prognosis or Diagnosis) reporting guidelines (Checklist 1) and performed all analyses in R (version 4.2.3; R Foundation for Statistical Computing) with relevant extension packages [55].

### Ethical Considerations

This study was approved by the Vanderbilt University Medical Center Institutional Review Board (220644), and data were deidentified.

## Results

### Cohort Characteristics

The full cohort of patient encounters was split temporally, based on the number of HAPIs, into model development and validation cohorts. The characteristics for each cohort are provided in Table 2. Among the model development cohort, those who developed HAPIs were older and male. Table 3 provides the model development cohort characteristics divided by whether a HAPI occurred.

**Table 2.** Characteristics for model development and validation cohorts. Measures were first taken during the hospital stay unless specified otherwise. Race and ethnicity were not included as candidate features.

			Development cohort (n=161,816 encounters)	Validation cohort (n=36,095 encounters)
Age (years), median (IQR)			56 (37-69)	56 (37-69)
Female, n (%)			84,727 (52.4)	17,060 (47.3)
**Race, n (%)**				
	White		125,322 (77.4)	27,649 (76.6)
	African American		26,299 (16.3)	5659 (15.7)
	Asian		2325 (1.4)	480 (1.3)
	American Indian or Alaska Native		289 (0.2)	66 (0.2)
	Pacific Islander		104 (0.1)	25 (0.1)
	Multiple		1185 (0.7)	231 (0.6)
Hispanic, n (%)			7406 (4.6)	2074 (5.7)
**Physiological and clinical features**				
	Temperature (°C), median (IQR)		36.7 (36.5-36.9)	36.7 (36.5-36.9)
	Respiratory rate (breaths per minute), median (IQR)		18 (16-19)	18 (16-20)
	Heart rate (beats per minute), median (IQR)		87 (74-100)	87 (75-101)
	BMI (kg/m^2^), median (IQR)		28.2 (24.1-33.4)	28.3 (24.2-33.5)
	Oxygen saturation (%), median (IQR)		98 (96-99)	98 (96-99)
	Systolic blood pressure (mm Hg), median (IQR)		131 (117-147)	130 (117-146)
	Diastolic blood pressure (mm Hg), median (IQR)		77 (68-88)	77 (67-87)
	Emergency department admissions, n (%)		91,363 (56.5)	20,972 (58.1)
	Intensive care admissions, n (%)		34,190 (21.1)	7831 (21.7)
	Length of stay (days), median (IQR)		4 (2-6)	4 (2-7)
	Smokers, n (%)		56,750 (35.1)	12,561 (34.8)
	Edema, n (%)		86,582 (53.5)	19,846 (55)
	Spinal cord injury, n (%)		5908 (3.7)	1428 (4)
	Dialysis, n (%)		92 (0.1)	74 (0.2)
	Tracheostomy, n (%)		2122 (1.3)	520 (1.4)
	Gastric tube, n (%)		35 (0)	5 (0)
	Central line, n (%)		20,648 (12.8)	4803 (13.3)
	Chest tube, n (%)		5186 (3.2)	1278 (3.5)
	Ostomy, n (%)		2059 (1.3)	459 (1.3)
	Drain, n (%)		17,800 (11)	4005 (11.1)
	ECMO^a^, n (%)		414 (0.3)	71 (0.2)
**Laboratory results, median (IQR)**				
	Hemoglobin A_1C_ (%)		6.1 (5.5-7.5)	6.1 (5.6-7.5)
	Hemoglobin (g/dL)		12.0 (10.3-13.6)	11.9 (10.2-13.5)
	Hematocrit (%)		36.0 (32.0-41.0)	36.0 (32.0-40.0)
	MCHC^b^ (g/dL)		33.0 (32.0-34.0)	32.9 (31.9-33.9)
	Red cell distribution width (%)		13.9 (13.0-15.5)	14.0 (13.0-15.6)
	Platelet count (×10^9^/L)		228 (174-291)	234 (179-298)
	Chloride (mEq/L)		105 (101-108)	104 (101-107)
	Lactate (mmol/L)		1.1 (0.8-1.9)	1.2 (0.8-2.0)
	Albumin (g/dL)		3.6 (3.1-4.0)	3.5 (3.0-3.9)
	Urine blood urea nitrogen		412 (260-603)	415 (275-609)
	Creatinine (mg/dL)		0.9 (0.9-1.3)	0.9 (0.8-1.3)
	Glucose (mmol/L)		114 (96-146)	114 (96-145)
**Nursing assessment features**				
	Braden scale score, median (IQR)		20 (18-22)	20 (17-21)
	Level of consciousness=2, n (%)		21,357 (13.2)	5043 (14)
	Gait transfer=20, n (%)		10,673 (6.6)	2190 (6.1)
	Glasgow Coma Scale=3, n (%)		4872 (3)	961 (2.7)
	Malnutrition score=5, n (%)		1241 (0.8)	345 (1)
**Outcomes, n (%)**				
	Any pressure injury		9259 (5.7)	2143 (5.9)
	Hospital-acquired pressure injury		4362 (2.7)	1096 (3)

a ECMO: extracorporeal membrane oxygenation.

b MCHC: mean corpuscular hemoglobin concentration.

**Table 3.** Model development cohort characteristics with and without hospital acquired pressure injury. Measures were the first taken during the hospital stay unless specified otherwise. Race and ethnicity were not included as candidate features.

		No hospital-acquired pressure injury (n=157,454 encounters)	Hospital-acquired pressure injury (n=4362 encounters)
Age (years), median (IQR)		56 (37-68)	64 (52-74)
Female, n (%)		82,999 (52.7)	1728 (39.6)
**Race, n (%)**			
	White	121,786 (77.3)	3536 (81.1)
	African American	25,654 (16.3)	645 (14.8)
	Asian	2290 (1.5)	35 (0.8)
	American Indian or Alaska Native	285 (0.2)	4 (0.1)
	Pacific Islander	102 (0.1)	2 (0)
	Multiple	1154 (0.7)	31 (0.7)
Hispanic, n (%)		7306 (4.6)	100 (2.3)
**Physiologic and clinical features**			
	Temperature (°C), median (IQR)	36.7 (36.5-36.9)	36.7 (36.4-37.0)
	Respiratory rate (breaths per minute), median (IQR)	18.0 (16.0-19.0)	18.0 (16.0-22.0)
	Heart rate (beats per minute), median (IQR)	87.0 (74.0-100.0)	91.0 (77.0-106.0)
	BMI (kg/m^2^), median (IQR)	28.2 (24.1-33.5)	26.8 (22.6-32.2)
	Oxygen saturation (%), median (IQR)	98.0 (96.0-99.0)	97.0 (95.0-99.0)
	Systolic blood pressure (mm Hg), median (IQR)	131.0 (117.0-147.0)	124.0 (107.0-142.0)
	Diastolic blood pressure (mm Hg), median (IQR)	78.0 (68.0-88.0)	72.0 (61.0-84.0)
	Emergency department admissions, n (%)	88,552 (56.2)	2811 (64.4)
	Intensive care admissions, n (%)	31,795 (20.2)	2395 (54.9)
	Length of stay (days), median (IQR)	3 (2-6)	15 (8-26)
	Smokers, n (%)	55,278 (35.1)	1472 (33.7)
	Edema, n (%)	82,640 (52.5)	3942 (90.4)
	Spinal cord injury, n (%)	5398 (3.4)	510 (11.7)
	Dialysis, n (%)	81 (0.1)	11 (0.3)
	Tracheostomy, n (%)	1491 (0.9)	631 (14.5)
	Gastric tube, n (%)	24 (0)	11 (0.3)
	Central line, n (%)	18,350 (11.7)	2298 (52.7)
	Chest tube, n (%)	4598 (2.9)	588 (13.5)
	Ostomy, n (%)	1881 (1.2)	178 (4.1)
	Drain, n (%)	16,888 (10.7)	912 (20.9)
	ECMO^a^, n (%)	242 (0.2)	172 (3.9)
**Laboratory results, median (IQR)**			
	Hemoglobin A_1C_ (%)	6.1 (5.5-7.5)	6.0 (5.4-7.1)
	Hemoglobin (g/dL)	12.0 (10.3-13.6)	12.0 (10.3-13.6)
	Hematocrit (%)	37.0 (32.0-41.0)	34.0 (29.0-40.0)
	MCHC^b^ (g/dL)	33.0 (32.0-34.0)	32.6 (31.5-33.7)
	Red cell distribution width (%)	13.9 (13.0-15.5)	14.9 (13.5-16.8)
	Platelet count (×10^9^/L)	228.0 (175.0-291.0)	215.0 (151.0-298.0)
	Chloride (mEq/L)	105.0 (101.0-108.0)	104.0 (99.0-108.0)
	Lactate (mmol/L)	1.1 (0.8-1.3)	1.4 (0.9-2.5)
	Albumin (g/dL)	3.6 (3.1-4.0)	3.1 (2.6-3.5)
	Urine blood urea nitrogen	412.0 (263.0-603.0)	410.0 (244.0-605.5)
	Creatinine (mg/dL)	0.9 (0.8-1.3)	1.2 (0.8-1.9)
	Glucose (mmol/L)	114.0 (96.0-145.0)	125.0 (101.0-168.0)
**Nursing assessment features**			
	Braden scale score, median (IQR)	20.0 (18.0-22.0)	15.0 (13.0-18.0)
	Level of consciousness=2, n (%)	20,712 (13.2)	645 (14.8)
	Gait transfer=20, n (%)	10,055 (6.4)	618 (14.2)
	Glasgow Coma Scale=3, n (%)	4406 (2.8)	466 (10.7)
	Malnutrition score=5, n (%)	1166 (0.7)	75 (1.7)

a ECMO: extracorporeal membrane oxygenation.

b MCHC: mean corpuscular hemoglobin concentration.

### Model Description

**Figure 3.**
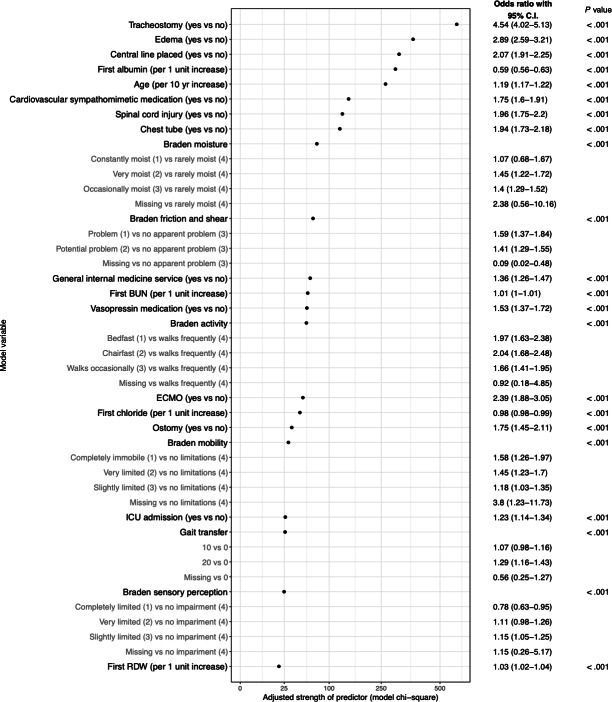
Relative importance of features used in the final Vanderbilt model. Gray subfeatures represent item comparisons used to generate features. *P* values for variable significance were derived using the Wald *χ*^2^ test. BUN: blood urea nitrogen; ECMO: extracorporeal membrane oxygenation; ICU: intensive care unit; RDW: red cell distribution width.

We determined 22 features were necessary to achieve a parsimonious yet highly accurate model. Again, features were selected using a LASSO approach. We fit the final model with 4362 HAPI encounters and 291 degrees of freedom, which indicated the model was unlikely to overfit the data. Of the 40 features that exhibited association with developing a HAPI, the top 5 features included tracheostomy (odds ratio [OR] 4.5, 95% CI 4.0-5.1), peripheral edema (OR 2.9, 95% CI 2.6-3.2), central line (OR 2.1, 95% CI 1.9-2.3), first albumin measure (OR 0.6, 95% CI 0.6-0.6), and age (OR 1.2, 95% CI 1.2-1.2) (Figure 3). Although the directionality for each feature may vary, the relative importance in Figure 3 was ranked on a single scale. Additional significant features included whether the patient was on sympathomimetic medications, had a spinal cord injury or chest tube, and individual Braden score component measures. The final Vanderbilt model with 22 features provided excellent discriminatory ability with an AUC of 0.897 (95% CI 0.893-0.901). Multimedia Appendix 2 depicts the probability density plot for the development and validation cohorts.

### Comparison With the Braden Scale

**Figure 4.**
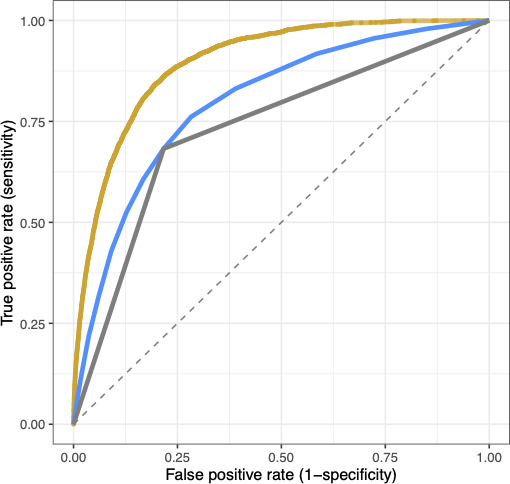
Area under the receiver operating characteristic curve comparing the Vanderbilt (gold), continuous Braden (blue), and dichotomous Braden (gray) models.

Using the model development cohort, the Vanderbilt model achieved an AUC of 0.897 (95% CI 0.893-0.901), compared to 0.798 (95% CI 0.791-0.803) and 0.733 (95% CI 0.725-0.740) for the continuous and dichotomous Braden, respectively (Figure 4).

### Model Validation

**Figure 5.**
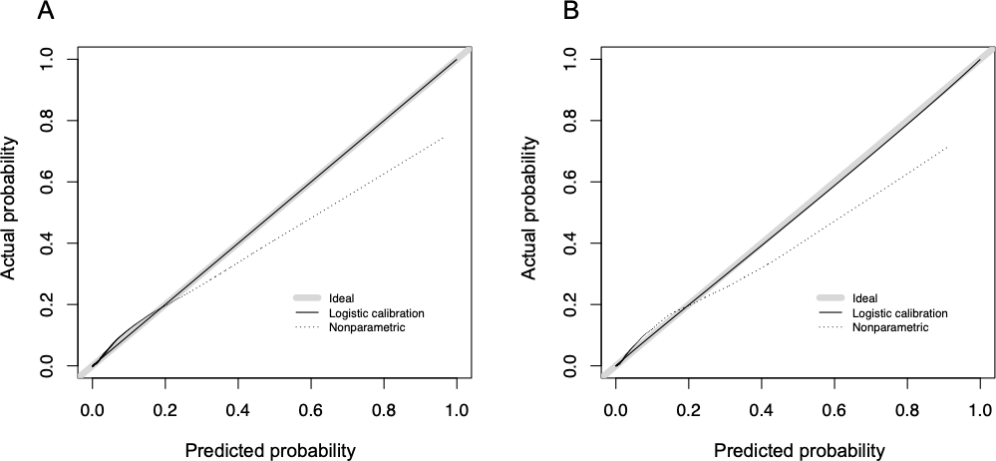
Calibration curves for model development (left) and validation (right). Logistic calibration (solid line) represents parameter-based calibration (logistic regression model fit between predicted and observed values). Nonparametric calibration (dotted line) represents locally estimated scatterplot smoothing trend between predicted and observed values.

The validation cohort consisted of 34,999 hospitalizations without a HAPI and 1096 hospitalizations with at least one HAPI. Model development and validation cohorts were compared to confirm that each had similar characteristics. Overall, characteristics were similar between the 2 cohorts (Table 3). We applied the same model from the development cohort to the validation cohort without adjusting coefficients, which provided a concordance statistic of 0.893 (95% CI 0.885-0.899; Table 4). Model calibration was consistent between the development and validation cohorts. The calibration curve indicated the model most accurately predicted risk for patients in the range of 0%-25% predicted risk (Figure 5); above this, the model could overpredict a HAPI. Since the model was intended to bring nurse attention and interventions to patients who would otherwise be overlooked, we believe the miscalibration at higher percentages was less clinically relevant. There was no evidence of collinearity. We are confident that this model performs well for most patients across the intensive care and general hospital settings, as 98.2% of the cohort had a predicted risk of less than 25%.

Since the model was designed to be used broadly in the general adult hospital, we performed a post hoc analysis among subpopulations for age (older than 65 years), gender, race, ethnicity, intensive care unit admission, and Braden score (greater than 18). The subpopulation analysis revealed only slight changes in discrimination performance (Multimedia Appendix 3).

To operationalize the Vanderbilt model in the EHR (Epic), we generated the equation below. The output from the equation is a numeric probability from 0 to 1. Z is the sum of –4.1812002 and the product of the coefficient and measured value (eg, first albumin) for each feature. In Multimedia Appendix 4, we provide the coefficients for the equation. The model has been deployed as a population management tool to generate risk prediction data at Vanderbilt University Medical Center, but the output is only available for the research team until a trial period has been completed and governance has approved it for patient care. Within a report for multiple patients, output from the model is available as a column among other relevant factors to prioritize pressure injury interventions. As part of the implementation plan, we have created an application for potential users to test the model [56].


$$Probability\;of\;hospital-acquired\;pressure\;injury=\frac{1}{\left(1+exp\left(-Z\right)\right)}$$

**Table 4.** Prediction model performance for hospital-acquired pressure injury.

Model	Area under the curve (95% CI)	Brier score	Integrated calibration index	Intercept	Slope
Vanderbilt (logistic regression)	0.893 (0.885-0.899)	0.026	0.006	−0.041	0.977
Continuous Braden (logistic regression)	0.799 (0.789-0.811)	0.028	0.006	0.178	1.034
Dichotomous Braden (score<18)	0.733 (0.725-0.740)	0.025	Too few levels to compute	0.0	1.0

## Discussion

### Principal Findings

We developed and validated a risk prediction model for HAPIs that can be used in the general adult population. The model achieved excellent discrimination and adequate calibration (Table 4). Although several recent models have achieved similar performance, our model may have the greatest likelihood of reducing HAPIs because it was built with the foresight of overcoming known barriers to implementation of risk-prediction clinical decision support (Figure 1). According to the scoring criteria in Figure 1, the present model would have achieved 8 of a possible 10, compared to the current highest score of 6. It lost points for being limited to adults from a single institution (broadly applicable) and partially specified intervention (actionable criteria). Limiting development of the model to a single institution could limit the generalizability due to documentation patterns and data availability. Although we specified how to deploy the model in the EHR, the intervention components and implementation strategies were underspecified for implementation and evaluation. The next step is to test the effectiveness of the model in a pragmatic randomized clinical trial in which the intervention will be fully specified [57].

Although our model achieved similar performance and used the same regression approach as the top 3 models in Figure 1 (Ladios-Martin et al [25], Levy et al [27], and Song et al [26]), many of the most important features among the models varied. Among the most important features in the Ladios-Martin et al [25] model (eg, medical service, days of antidiabetic therapy, ability to eat, number of red blood cell units transfused, and hemoglobin range), only medical service was similar to our model. Relatedly, 2 important features in the Levy et al [27] model overlapped (friction and mobility). However, several important features from the Song et al [26] model (albumin, gait/transferring, activity, blood urea nitrogen, chloride, and spinal cord injury) overlapped with our model. We anticipate the similarity in features between our model and the Song et al [26] model was due to use of the same EHR and the models being developed at academic medical centers in the United States.

Limited implementation of risk prediction models in the EHR presents a critical challenge in health care today; the barrier is now less about the performance of risk prediction models and more the sociotechnical obstacles to uptake in patient care [58-60]. Despite the growing availability and sophistication of these models, their integration into routine clinical practice remains inadequate. Of the 22 models identified, we were unable to find one that decreased HAPIs. Even when prespecified elements for an implementable model are fulfilled, concerted efforts are needed from various stakeholders. Collaboration between health care organizations, technology developers, and regulatory bodies is essential to establish standards and guidelines for incorporating risk prediction models into EHR systems [61]. Enhancing data infrastructure, promoting data standardization, and developing robust privacy and security frameworks are crucial steps toward facilitating the implementation of these models [62]. Additionally, targeted education and training initiatives can help build trust and confidence among health care providers, encouraging their acceptance and use of risk prediction models in clinical practice, along with actionable steps to take for patients at highest risk [63,64]. Furthermore, there are significant socio-organizational barriers that impede the implementation of risk prediction models in EHRs. Resistance to change, lack of awareness or understanding among health care providers, and concerns regarding liability and accountability are common challenges faced by health care institutions. Clinicians may be skeptical of relying on risk prediction models, fearing that their judgment and decision-making autonomy may be compromised. The integration of risk prediction models also requires extensive training and education for health care providers, which may be resource-intensive and time-consuming [65,66]. Only when these barriers are addressed in a pragmatic manner can risk-prediction clinical decision support models improve patient outcomes.

Pragmatic trials are crucial in testing the real-world effectiveness and utility of interventions in health care settings [57,67,68]. These trials provide valuable insights into how interventions perform when integrated into routine clinical practice, considering factors such as patient outcomes, workflow integration, and usability. Institutions are beginning to develop the infrastructure and stakeholder engagement to support pragmatic trials. At our institution, Semler and colleagues [69] tested the effectiveness of balanced crystalloids and saline for fluids in critically ill adults. This pragmatic trial was cluster-randomized with 5 intensive care units. The authors found that use of balanced crystalloids resulted in a lower rate of death. A key aspect that makes pragmatic trials feasible is the use of existing infrastructure and real-world practice, which typically includes an inclusive patient population, minimal staff training, flexible protocols, minimally disruptive interventions, and outcomes captured as part of care. For pressure injuries specifically, the intervention infrastructure and guidance already exist as part of routine care; however, risk prediction will help identify and prioritize the most at-risk patients for targeted intervention. Preliminarily, we envision a clinician will use a list of patients ranked highest to lowest risk for HAPI.

### Strengths and Limitations

Pressure injury prediction models have shown promise in identifying individuals at risk of developing pressure injuries. However, there are several limitations with these models, including ours, that should be considered. First, documentation of pressure injuries varies by institution and can lead to misclassification. We found that documentation of some pressure injuries carried over from previous encounters. On further testing, we found that missing measures (eg, albumin) can lead to inaccurate prediction. Thus, we chose to use a replicable imputation method with the median. Although our prediction model was developed and validated using incident HAPIs, documentation errors should be carefully considered. To increase the generalizability of our model, we chose not to include text from notes, despite evidence that use of clinical notes may have predictive power. Although we had a relatively large sample size that was sufficient to include all important features, the patient cohort was from a single institution and may not generalize to institutions in different geographical areas or using different EHRs. Finally, we chose to use an interpretable model that could be operationalized in current EHRs; however, other models may provide slightly higher performance. We anticipate certain EHR vendors will continue to develop capabilities for implementing complex machine learning models for more complicated prediction tasks. In anticipation of this, we performed a preliminary analysis of random forest, generalized additive model, and XGBoost. Of these models, we found that XGBoost had higher discrimination than ours in the model development cohort (AUC 0.960, 95% CI 0.957-0.962 vs AUC 0.893, 95% CI 0.885-0.899). In the model validation cohort, however, performance was not superior to logistic regression (AUC 0.869, 95% CI 0.861-0.877 vs AUC 0.893, 95% CI 0.885-0.899). Future work is needed to fully optimize the machine learning models and explore the tradeoff between interpretability and performance.

### Conclusion

Despite numerous models developed to predict pressure injuries, studies demonstrating improved patient outcomes are missing. This is because implementing risk prediction models for routine patient care is complex and requires model developers, clinicians, and researchers to address challenges early in the process. Therefore, we developed and validated an accurate prediction model for HAPIs that fulfilled necessary elements for implementation. The next step is to overcome socio-organizational barriers to rigorously evaluate the model through a pragmatic randomized clinical trial that includes targeted intervention for patients at highest risk. Our approach to developing an implementable risk prediction model, with feasible plans to evaluate its effectiveness, is generalizable to risk prediction and may be necessary to unlock the potential of this technology and improve decision-making.

## Supplementary material

10.2196/51842 Multimedia Appendix 1 Full cohort characteristics.

10.2196/51842 Multimedia Appendix 2 Density and probability plots for model development (A) and validation (B).

10.2196/51842 Multimedia Appendix 3 Subpopulation analysis of adult general hospital patients.

10.2196/51842 Multimedia Appendix 4 Features and coefficients of model and equation.

10.2196/51842 Checklist 1 Transparent Reporting of a Multivariable Prediction Model for Individual Prognosis or Diagnosis (TRIPOD) checklist.

## Abbreviations

AUC: area under the receiver operating curve
EHR: electronic health record
HAPI: hospital-acquired pressure injury
LASSO: least absolute shrinkage and selection operator
OR: odds ratio
TRIPOD: Transparent Reporting of a Multivariable Prediction Model for Individual Prognosis or Diagnosis
