# Supplementary material for: Implementable Prediction of Pressure Injuries in Hospitalized Adults: Model Development and Validation
Source: JMIR Med Inform. 2024 May 8;12:e51842. doi: 10.2196/51842 (PMC11094428; doi:10.2196/51842)
Supplement: Multimedia Appendix 1 [file medinform-v12-e51842-s001.docx]

| **Multimedia Appendix 1. Full cohort characteristics** | | | |
| --- | --- | --- | --- |
| **Age** |  |  |  |
| Q1/Median/Q3 [Mean (SD)] | 37/56/69 [53.7 (18.7)] | 37/56/69 [53.6 (19)] | 37/56/69 [53.7 (18.8)] |
| **Gender** |  |  |  |
| Female | 84727 (52.4%) | 19032 (52.7%) | 103759 (52.4%) |
| Male | 77076 (47.6%) | 17060 (47.3%) | 94136 (47.6%) |
| Unknown | 13 (0.0%) | 3 (0.0%) | 16 (0.0%) |
| **Race** |  |  |  |
| American Indian | 289 (0.2%) | 66 (0.2%) | 355 (0.2%) |
| Asian | 2325 (1.4%) | 480 (1.3%) | 2805 (1.4%) |
| Black | 26299 (16.3%) | 5659 (15.7%) | 31958 (16.1%) |
| Multiple | 1185 (0.7%) | 231 (0.6%) | 1416 (0.7%) |
| Native Hawaiian | 104 (0.1%) | 25 (0.1%) | 129 (0.1%) |
| White | 125322 (77.4%) | 27649 (76.6%) | 152971 (77.3%) |
| Missing | 6292 (3.9%) | 1985 (5.5%) | 8277 (4.2%) |
| **Ethnicity** |  |  |  |
| Hispanic | 7406 (4.6%) | 2074 (5.7%) | 9480 (4.8%) |
| Not Hispanic | 151033 (93.3%) | 33070 (91.6%) | 184103 (93.0%) |
| Missing | 3377 (2.1%) | 951 (2.6%) | 4328 (2.2%) |
| **Insurance Status** |  |  |  |
| Government | 96514 (59.6%) | 21171 (58.7%) | 117685 (59.5%) |
| Other | 2413 (1.5%) | 806 (2.2%) | 3219 (1.6%) |
| Private | 53439 (33.0%) | 12264 (34.0%) | 65703 (33.2%) |
| Missing | 9450 (5.8%) | 1854 (5.1%) | 11304 (5.7%) |
| Admitted Through ED |  |  |  |
| 0 | 70453 (43.5%) | 15123 (41.9%) | 85576 (43.2%) |
| 1 | 91363 (56.5%) | 20972 (58.1%) | 112335 (56.8%) |
| ICU Admission |  |  |  |
| 0 | 127626 (78.9%) | 28264 (78.3%) | 155890 (78.8%) |
| 1 | 34190 (21.1%) | 7831 (21.7%) | 42021 (21.2%) |
| Length of Stay |  |  |  |
| Q1/Median/Q3 [Mean (SD)] | 2/4/6 [5.6 (7.1)] | 2/4/7 [6.3 (8.5)] | 2/4/7 [5.7 (7.3)] |
| Smoking Status |  |  |  |
| 0 | 104485 (64.6%) | 23392 (64.8%) | 127877 (64.6%) |
| 1 | 56750 (35.1%) | 12561 (34.8%) | 69311 (35.0%) |
| Missing | 581 (0.4%) | 142 (0.4%) | 723 (0.4%) |
| Temperature |  |  |  |
| Q1/Median/Q3 [Mean (SD)] | 97.7/98.1/98.5 [98.1 (1)] | 97.7/98.1/98.5 [98.1 (1.3)] | 97.7/98.1/98.5 [98.1 (1.1)] |
| Missing | 867 (0.5%) | 273 (0.8%) | 1140 (0.6%) |
| Respiratory Rate |  |  |  |
| Q1/Median/Q3 [Mean (SD)] | 16/18/19 [18.3 (4.3)] | 16/18/20 [18.4 (4.2)] | 16/18/20 [18.3 (4.3)] |
| Missing | 186 (0.1%) | 38 (0.1%) | 224 (0.1%) |
| Heart Rate |  |  |  |
| Q1/Median/Q3 [Mean (SD)] | 74/87/100 [88.5 (20)] | 75/87/101 [88.9 (20.1)] | 74/87/101 [88.6 (20)] |
| Missing | 218 (0.1%) | 52 (0.1%) | 270 (0.1%) |
| BMI |  |  |  |
| Q1/Median/Q3 [Mean (SD)] | 24.1/28.2/33.4 [29.5 (7.9)] | 24.2/28.3/33.5 [29.6 (7.9)] | 24.1/28.2/33.4 [29.5 (7.9)] |
| Missing | 38996 (24.1%) | 7707 (21.4%) | 46703 (23.6%) |
| Oxygen Saturation |  |  |  |
| Q1/Median/Q3 [Mean (SD)] | 96/98/99 [97.1 (3.4)] | 96/98/99 [97.1 (3.6)] | 96/98/99 [97.1 (3.5)] |
| Missing | 1170 (0.7%) | 118 (0.3%) | 1288 (0.7%) |
| Systolic BP |  |  |  |
| Q1/Median/Q3 [Mean (SD)] | 117/131/147 [132.7 (23.6)] | 117/130/146 [132.2 (23.8)] | 117/131/147 [132.7 (23.6)] |
| Missing | 732 (0.5%) | 149 (0.4%) | 881 (0.4%) |
| Diastolic BP |  |  |  |
| Q1/Median/Q3 [Mean (SD)] | 68/77/88 [77.8 (16)] | 67/77/87 [77.6 (16.2)] | 67/77/88 [77.8 (16)] |
| Missing | 732 (0.5%) | 149 (0.4%) | 881 (0.4%) |
| Hemoglobin |  |  |  |
| Q1/Median/Q3 [Mean (SD)] | 10.3/12/13.6 [11.9 (2.4)] | 10.2/11.9/13.5 [11.8 (2.4)] | 10.3/12/13.6 [11.9 (2.4)] |
| Missing | 10806 (6.7%) | 2028 (5.6%) | 12834 (6.5%) |
| Hemoglobin A1C |  |  |  |
| Q1/Median/Q3 [Mean (SD)] | 5.5/6.1/7.5 [6.8 (2.1)] | 5.6/6.1/7.5 [6.8 (2)] | 5.5/6.1/7.5 [6.8 (2.1)] |
| Missing | 124048 (76.7%) | 27904 (77.3%) | 151952 (76.8%) |
| Hematocrit |  |  |  |
| Q1/Median/Q3 [Mean (SD)] | 32/36/41 [36.1 (6.7)] | 32/36/40 [35.9 (6.8)] | 32/36/41 [36.1 (6.7)] |
| Missing | 7372 (4.6%) | 1483 (4.1%) | 8855 (4.5%) |
| MCHC |  |  |  |
| Q1/Median/Q3 [Mean (SD)] | 32/33/34 [32.9 (1.6)] | 31.9/32.9/33.9 [32.8 (1.6)] | 32/33/34 [32.9 (1.6)] |
| Missing | 10933 (6.8%) | 2108 (5.8%) | 13041 (6.6%) |
| RDW |  |  |  |
| Q1/Median/Q3 [Mean (SD)] | 13/13.9/15.5 [14.6 (2.4)] | 13/14/15.6 [14.6 (2.5)] | 13/13.9/15.6 [14.6 (2.4)] |
| Missing | 11029 (6.8%) | 2139 (5.9%) | 13168 (6.7%) |
| Platelet Count |  |  |  |
| Q1/Median/Q3 [Mean (SD)] | 174/228/291 [241.3 (112)] | 179/234/298 [247.4 (115.6)] | 175/229/292 [242.4 (112.7)] |
| Missing | 8677 (5.4%) | 1619 (4.5%) | 10296 (5.2%) |
| Chloride |  |  |  |
| Q1/Median/Q3 [Mean (SD)] | 101/105/108 [104.2 (5.5)] | 101/104/107 [103.8 (5.6)] | 101/105/107 [104.1 (5.5)] |
| Missing | 22560 (13.9%) | 4960 (13.7%) | 27520 (13.9%) |
| BUN |  |  |  |
| Q1/Median/Q3 [Mean (SD)] | 11/16/24 [21.4 (18.4)] | 11/16/24 [21.5 (18.9)] | 11/16/24 [21.4 (18.5)] |
| Missing | 22568 (13.9%) | 4966 (13.8%) | 27534 (13.9%) |
| Creatinine |  |  |  |
| Q1/Median/Q3 [Mean (SD)] | 0.8/0.9/1.3 [1.5 (1.8)] | 0.8/0.9/1.3 [1.5 (1.9)] | 0.8/0.9/1.3 [1.5 (1.9)] |
| Missing | 19929 (12.3%) | 4488 (12.4%) | 24417 (12.3%) |
| Lactate |  |  |  |
| Q1/Median/Q3 [Mean (SD)] | 0.8/1.1/1.9 [1.8 (2)] | 0.8/1.2/2 [1.9 (2.2)] | 0.8/1.1/1.9 [1.8 (2.1)] |
| Missing | 131057 (81.0%) | 28573 (79.2%) | 159630 (80.7%) |
| Albumin |  |  |  |
| Q1/Median/Q3 [Mean (SD)] | 3.1/3.6/4 [3.5 (0.6)] | 3/3.5/3.9 [3.4 (0.6)] | 3.1/3.6/4 [3.5 (0.6)] |
| Missing | 74882 (46.3%) | 16795 (46.5%) | 91677 (46.3%) |
| Urine BUN |  |  |  |
| Q1/Median/Q3 [Mean (SD)] | 260/412/603 [463.6 (273.2)] | 275/415/609 [468.9 (267.6)] | 264/413/604 [464.8 (272)] |
| Missing | 155002 (95.8%) | 34146 (94.6%) | 189148 (95.6%) |
| Glucose |  |  |  |
| Q1/Median/Q3 [Mean (SD)] | 96/114/146 [135.9 (80)] | 96/114/145 [135.5 (81.5)] | 96/114/146 [135.8 (80.3)] |
| Missing | 21461 (13.3%) | 4803 (13.3%) | 26264 (13.3%) |
| Braden Score |  |  |  |
| Q1/Median/Q3 [Mean (SD)] | 18/20/22 [19.3 (3.2)] | 17/20/21 [19 (3.1)] | 18/20/22 [19.3 (3.1)] |
| Missing | 2950 (1.8%) | 985 (2.7%) | 3935 (2.0%) |
| Braden-Nutrition |  |  |  |
| 1 | 2622 (1.6%) | 592 (1.6%) | 3214 (1.6%) |
| 2 | 30148 (18.6%) | 7740 (21.4%) | 37888 (19.1%) |
| 3 | 95339 (58.9%) | 21897 (60.7%) | 117236 (59.2%) |
| 4 | 30673 (19.0%) | 4866 (13.5%) | 35539 (18.0%) |
| Missing | 3034 (1.9%) | 1000 (2.8%) | 4034 (2.0%) |
| Braden-Mobility |  |  |  |
| 1 | 3276 (2.0%) | 702 (1.9%) | 3978 (2.0%) |
| 2 | 19884 (12.3%) | 4639 (12.9%) | 24523 (12.4%) |
| 3 | 67756 (41.9%) | 16623 (46.1%) | 84379 (42.6%) |
| 4 | 67871 (41.9%) | 13135 (36.4%) | 81006 (40.9%) |
| Missing | 3029 (1.9%) | 996 (2.8%) | 4025 (2.0%) |
| Braden-Activity |  |  |  |
| 1 | 27064 (16.7%) | 6930 (19.2%) | 33994 (17.2%) |
| 2 | 11605 (7.2%) | 2737 (7.6%) | 14342 (7.2%) |
| 3 | 69518 (43.0%) | 16969 (47.0%) | 86487 (43.7%) |
| 4 | 50704 (31.3%) | 8476 (23.5%) | 59180 (29.9%) |
| Missing | 2925 (1.8%) | 983 (2.7%) | 3908 (2.0%) |
| Braden-Moisture |  |  |  |
| 1 | 247 (0.2%) | 58 (0.2%) | 305 (0.2%) |
| 2 | 1816 (1.1%) | 392 (1.1%) | 2208 (1.1%) |
| 3 | 30482 (18.8%) | 7103 (19.7%) | 37585 (19.0%) |
| 4 | 126307 (78.1%) | 27561 (76.4%) | 153868 (77.7%) |
| Missing | 2964 (1.8%) | 981 (2.7%) | 3945 (2.0%) |
| Braden-Friction and Shear |  |  |  |
| 1 | 3488 (2.2%) | 715 (2.0%) | 4203 (2.1%) |
| 2 | 34878 (21.6%) | 8173 (22.6%) | 43051 (21.8%) |
| 3 | 120476 (74.5%) | 26214 (72.6%) | 146690 (74.1%) |
| Missing | 2974 (1.8%) | 993 (2.8%) | 3967 (2.0%) |
| Braden-Sensory Perceptions |  |  |  |
| 1 | 2610 (1.6%) | 583 (1.6%) | 3193 (1.6%) |
| 2 | 6538 (4.0%) | 1338 (3.7%) | 7876 (4.0%) |
| 3 | 31786 (19.6%) | 7450 (20.6%) | 39236 (19.8%) |
| 4 | 117949 (72.9%) | 25743 (71.3%) | 143692 (72.6%) |
| Missing | 2933 (1.8%) | 981 (2.7%) | 3914 (2.0%) |
| Consciousness |  |  |  |
| 0 | 4041 (2.5%) | 1022 (2.8%) | 5063 (2.6%) |
| 1 | 27368 (16.9%) | 6323 (17.5%) | 33691 (17.0%) |
| 2 | 21357 (13.2%) | 5043 (14.0%) | 26400 (13.3%) |
| Missing | 109050 (67.4%) | 23707 (65.7%) | 132757 (67.1%) |
| Gait Transfer |  |  |  |
| 0 | 111523 (68.9%) | 24847 (68.8%) | 136370 (68.9%) |
| 10 | 38426 (23.7%) | 8680 (24.0%) | 47106 (23.8%) |
| 20 | 10673 (6.6%) | 2190 (6.1%) | 12863 (6.5%) |
| Missing | 1194 (0.7%) | 378 (1.0%) | 1572 (0.8%) |
| Glasgow Coma Score |  |  |  |
| 3 | 4872 (3.0%) | 961 (2.7%) | 5833 (2.9%) |
| 4 | 279 (0.2%) | 54 (0.1%) | 333 (0.2%) |
| 5 | 308 (0.2%) | 73 (0.2%) | 381 (0.2%) |
| 6 | 875 (0.5%) | 157 (0.4%) | 1032 (0.5%) |
| 7 | 862 (0.5%) | 162 (0.4%) | 1024 (0.5%) |
| 8 | 826 (0.5%) | 157 (0.4%) | 983 (0.5%) |
| 9 | 842 (0.5%) | 184 (0.5%) | 1026 (0.5%) |
| 10 | 1457 (0.9%) | 325 (0.9%) | 1782 (0.9%) |
| 11 | 1624 (1.0%) | 359 (1.0%) | 1983 (1.0%) |
| 12 | 1168 (0.7%) | 226 (0.6%) | 1394 (0.7%) |
| 13 | 2521 (1.6%) | 559 (1.5%) | 3080 (1.6%) |
| 14 | 12114 (7.5%) | 2847 (7.9%) | 14961 (7.6%) |
| 15 | 128993 (79.7%) | 29110 (80.6%) | 158103 (79.9%) |
| Missing | 5075 (3.1%) | 921 (2.6%) | 5996 (3.0%) |
| Hopkins Mobility |  |  |  |
| (1) Bed - only lying | 0 (0%) | 874 (2.4%) | 874 (0.4%) |
| (2) Bed - turn self/bed activity | 1 (0.0%) | 2135 (5.9%) | 2136 (1.1%) |
| (3) Bed - sit at edge of bed | 0 (0%) | 348 (1.0%) | 348 (0.2%) |
| (4) Chair - transfer to chair | 0 (0%) | 501 (1.4%) | 501 (0.3%) |
| (5) Stand greater than or equal to 1 minute | 0 (0%) | 384 (1.1%) | 384 (0.2%) |
| (6) Walk 10+ steps | 0 (0%) | 2806 (7.8%) | 2806 (1.4%) |
| (7) Walk 25+ feet | 0 (0%) | 2925 (8.1%) | 2925 (1.5%) |
| (8) Walk 250+ feet | 0 (0%) | 1385 (3.8%) | 1385 (0.7%) |
| Missing | 161815 (100.0%) | 24737 (68.5%) | 186552 (94.3%) |
| Malnuturition Score |  |  |  |
| 0 | 70199 (43.4%) | 15397 (42.7%) | 85596 (43.2%) |
| 1 | 12508 (7.7%) | 2703 (7.5%) | 15211 (7.7%) |
| 2 | 6601 (4.1%) | 1928 (5.3%) | 8529 (4.3%) |
| 3 | 3395 (2.1%) | 1223 (3.4%) | 4618 (2.3%) |
| 4 | 1681 (1.0%) | 448 (1.2%) | 2129 (1.1%) |
| 5 | 1241 (0.8%) | 345 (1.0%) | 1586 (0.8%) |
| Missing | 66191 (40.9%) | 14051 (38.9%) | 80242 (40.5%) |
| Spinal Cord Injury Injury |  |  |  |
| 0 | 155908 (96.3%) | 34667 (96.0%) | 190575 (96.3%) |
| 1 | 5908 (3.7%) | 1428 (4.0%) | 7336 (3.7%) |
| Per. Dialysis Cath. |  |  |  |
| 0 | 161724 (99.9%) | 36021 (99.8%) | 197745 (99.9%) |
| 1 | 92 (0.1%) | 74 (0.2%) | 166 (0.1%) |
| Tracheostomy |  |  |  |
| 0 | 159694 (98.7%) | 35575 (98.6%) | 195269 (98.7%) |
| 1 | 2122 (1.3%) | 520 (1.4%) | 2642 (1.3%) |
| G-Tube |  |  |  |
| 0 | 161781 (100.0%) | 36090 (100.0%) | 197871 (100.0%) |
| 1 | 35 (0.0%) | 5 (0.0%) | 40 (0.0%) |
| Central Line |  |  |  |
| 0 | 141168 (87.2%) | 31292 (86.7%) | 172460 (87.1%) |
| 1 | 20648 (12.8%) | 4803 (13.3%) | 25451 (12.9%) |
| Chest Tube |  |  |  |
| 0 | 156630 (96.8%) | 34817 (96.5%) | 191447 (96.7%) |
| 1 | 5186 (3.2%) | 1278 (3.5%) | 6464 (3.3%) |
| Ostomy |  |  |  |
| 0 | 159757 (98.7%) | 35636 (98.7%) | 195393 (98.7%) |
| 1 | 2059 (1.3%) | 459 (1.3%) | 2518 (1.3%) |
| Drain |  |  |  |
| 0 | 144016 (89.0%) | 32090 (88.9%) | 176106 (89.0%) |
| 1 | 17800 (11.0%) | 4005 (11.1%) | 21805 (11.0%) |
| ECMO |  |  |  |
| 0 | 161402 (99.7%) | 36024 (99.8%) | 197426 (99.8%) |
| 1 | 414 (0.3%) | 71 (0.2%) | 485 (0.2%) |
| Edema |  |  |  |
| 0 | 75234 (46.5%) | 16249 (45.0%) | 91483 (46.2%) |
| 1 | 86582 (53.5%) | 19846 (55.0%) | 106428 (53.8%) |
| ASA Score |  |  |  |
| 2 | 2 (0.0%) | 0 (0%) | 2 (0.0%) |
| 3 | 14 (0.0%) | 0 (0%) | 14 (0.0%) |
| Missing | 161800 (100.0%) | 36095 (100%) | 197895 (100.0%) |
| Any Pressure Injury |  |  |  |
| 0 | 152557 (94.3%) | 33952 (94.1%) | 186509 (94.2%) |
| 1 | 9259 (5.7%) | 2143 (5.9%) | 11402 (5.8%) |
| Pressure Injury on Admission |  |  |  |
| 0 | 156151 (96.5%) | 34907 (96.7%) | 191058 (96.5%) |
| 1 | 5665 (3.5%) | 1188 (3.3%) | 6853 (3.5%) |
| Hospital-Acquired Pressure Injury |  |  |  |
| 0 | 157454 (97.3%) | 34999 (97.0%) | 192453 (97.2%) |
| 1 | 4362 (2.7%) | 1096 (3.0%) | 5458 (2.8%) |
