# Supplementary figures and images for: Implementable Prediction of Pressure Injuries in Hospitalized Adults: Model Development and Validation
Source: JMIR Med Inform. 2024 May 8;12:e51842. doi: 10.2196/51842 (PMC11094428; doi:10.2196/51842)

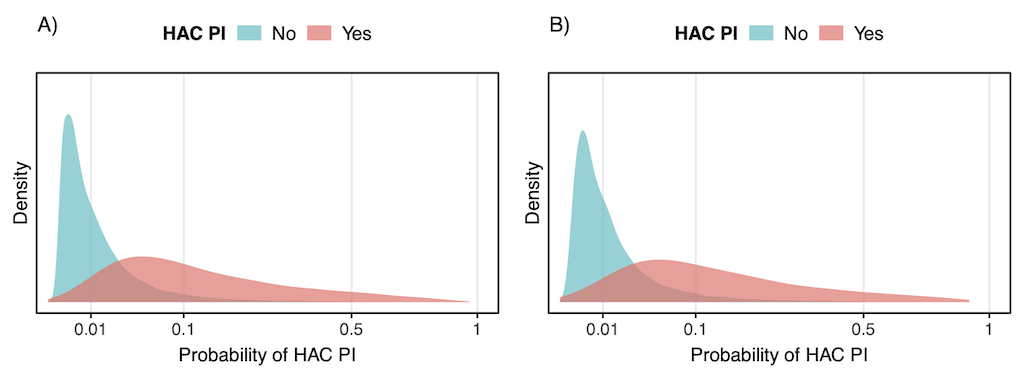

Supplement: Multimedia Appendix 2 [file medinform-v12-e51842-s002.png]
