# Supplementary material for: Implementable Prediction of Pressure Injuries in Hospitalized Adults: Model Development and Validation
Source: JMIR Med Inform. 2024 May 8;12:e51842. doi: 10.2196/51842 (PMC11094428; doi:10.2196/51842)
Supplement: Multimedia Appendix 3 [file medinform-v12-e51842-s003.docx]

| Multimedia Appendix 3. Subpopulation analysis of Adult General Hospital Patients. | | | | | |
| --- | --- | --- | --- | --- | --- |
|  |  |  | Development cohort |  | Validation cohort |
|  |  |  | AUC (95% CI) |  | AUC (95% CI) |
| Full Cohort |  |  | 0.897 (0.893 - 0.901) |  | 0.893 (0.885 - 0.899) |
| Age |  |  |  |  |  |
|  | < 65 |  | 0.924 (0.919 - 0.929) |  | 0.924 (0.912 - 0.934) |
|  | >= 65 |  | 0.839 (0.831 - 0.847) |  | 0.823 (0.809 - 0.838) |
| Gender |  |  |  |  |  |
|  | Male |  | 0.883 (0.878 - 0.889) |  | 0.871 (0.859 - 0.882) |
|  | Female |  | 0.909 (0.904 - 0.915) |  | 0.913 (0.901 - 0.923) |
|  | Unknown |  | 1 (1 – 1) |  | NA |
| Race |  |  |  |  |  |
|  | White |  | 0.89 (0.886 - 0.895) |  | 0.884 (0.874 - 0.893) |
|  | Black |  | 0.919 (0.91 - 0.927) |  | 0.91 (0.889 - 0.93) |
|  | Asian |  | 0.926 (0.874 - 0.972) |  | 0.976 (0.952 - 0.998) |
|  | Other |  | 0.919 (0.786 - 0.997) |  | 0.949 (0.888 - 0.991) |
|  | Multiple |  | 0.873 (0.824 - 0.917) |  | 0.892 (0.703 - 0.995) |
|  | Missing |  | 0.94 (0.92 - 0.957) |  | 0.949 (0.919 - 0.967) |
| Ethnicity |  |  |  |  |  |
|  | Hispanic |  | 0.946 (0.927 - 0.964) |  | 0.954 (0.913 - 0.978) |
|  | Non-Hispanic |  | 0.895 (0.891 - 0.898) |  | 0.889 (0.88 - 0.897) |
|  | Unknown |  | 0.891 (0.869 - 0.914) |  | 0.896 (0.856 - 0.931) |
| Intensive Care Unit |  |  |  |  |  |
|  | Admitted to ICU |  | 0.829 (0.821 - 0.838) |  | 0.84 (0.825 - 0.855) |
|  | Not Admitted to ICU |  | 0.898 (0.892 - 0.904) |  | 0.892 (0.882 - 0.904) |
| Braden Score |  |  |  |  |  |
|  | >= 18 |  | 0.894 (0.885 - 0.902) |  | 0.895 (0.88 - 0.91) |
|  | < 18 |  | 0.785 (0.777 – 0.793) |  | 0.783 (0.766 - 0.8) |
| COVID Status |  |  |  |  |  |
|  | COVID Absent |  | 0.897 (0.893 - 0.901) |  | 0.892 (0.884 - 0.90) |
|  | COVID Present |  | 0.886 (0.860 – 0.910) |  | 0.881 (0.838 - 0.911) |
