# Supplementary material for: Implementable Prediction of Pressure Injuries in Hospitalized Adults: Model Development and Validation
Source: JMIR Med Inform. 2024 May 8;12:e51842. doi: 10.2196/51842 (PMC11094428; doi:10.2196/51842)
Supplement: Multimedia Appendix 4 [file medinform-v12-e51842-s004.docx]

| **Multimedia Appendix 4: Features and coefficients of model and equation.** |
| --- |
| Probability of HAC PI = 1/(1 + exp(–**Z**)) |
| **Z =** –4.1812002 + |
| **0.0177999** * Patient Age + |
| **0.31090067** * (**1** if Patient in on General Internal Medicine Service, 0 otherwise) + |
| **-0.5281964** * First Albumin value recorded during stay (use value of **3.6** if no Albumin measured) + |
| **0.00529961** * First BUN value recorded during stay (use value of **16** if no BUN measured) + |
| **-0.0175021** * First Chloride value recorded during stay (use value of **105** if no Chloride measured) + |
| **0.02907249** * First RDW CV value recorded during stay (use value of **13.9** if no RDW CV measured) + |
| **0.45523305** * (**1** if First Braden Mobility Score = “1-Completely Immobile”, **0** Otherwise) + |
| **0.37061835** * (**1** if First Braden Mobility Score = “2-Very Limited”, **0** Otherwise) + |
| **0.16693425** * (**1** if First Braden Mobility Score = “3-Slightly Limited”, **0** Otherwise) + |
| **1.33510627** * (**1** if First Braden Mobility Score is missing or not measured, **0** Otherwise) + |
| **0.06386941** * (**1** if First Braden Moisture Score = “1-Constantly Moist”, **0** Otherwise) + |
| **0.37059476** * (**1** if First Braden Moisture Score = “2-Very Moist”, **0** Otherwise) + |
| **0.33684558** * (**1** if First Braden Moisture Score = “3-Occasionally Moist”, **0** Otherwise) + |
| **0.86599357** * (**1** if First Braden Moisture Score is missing or not measured, **0** Otherwise) + |
| **0.67739212** * (**1** if First Braden Activity Score = “1-Bedfast”, **0** Otherwise) + |
| **0.71491035** * (**1** if First Braden Activity Score = “2-Chairfast”, **0** Otherwise) + |
| **0.50653582** * (**1** if First Braden Activity Score = “3-Walks Occasionally”, **0** Otherwise) + |
| **-0.0813083** * (**1** if First Braden Activity Score is missing or not measured, **0** Otherwise) + |
| **0.46265013** * (**1** if First Braden Friction and Shear Score = “1-Problem”, **0** Otherwise) + |
| **0.34686801** * (**1** if First Braden Friction and Shear Score = “2-Potential Problem”, **0** Otherwise) + |
| **-2.4211435** * (**1** if First Braden Friction and Shear Score is missing or not measured, **0** Otherwise) + |
| **-0.2547595** * (**1** if First Braden Sensory Perception Score = “1-Completely Limited”, **0** Otherwise) + |
| **0.10338965** * (**1** if First Braden Sensory Perception Score = “2-Very Limited”, **0** Otherwise) + |
| **0.13860724** * (**1** if First Braden Sensory Perception Score = “3-Slightly Limited”, **0** Otherwise) + |
| **0.13982251** * (**1** if First Braden Sensory Perception Score is missing or not measured, **0** Otherwise) + |
| **0.25466541** * (**1** if First Gait Transfer Score = “20-Impaired Gait”, **0** Otherwise) + |
| **0.06334588** * (**1** if First Gait Transfer Score = “10-Weak Gait”, **0** Otherwise) + |
| **-0.585254** * (**1** if First Gait Transfer Score is missing or not measured, **0** Otherwise) + |
| **0.20985863** * (**1** if patient is admitted to an ICU, **0** otherwise) + |
| **0.67228617** * (**1** if patient has a spinal cord injury, **0** otherwise) + |
| **0.42814952** * (**1** if patient is on vasopressin medication, **0** otherwise) + |
| **0.55763892** * (**1** if patient is on cardio. sympathomimetic medication, **0** otherwise) + |
| **1.06047082** * (**1** if patient has edema present, **0** otherwise) + |
| **1.51259407** * (**1** if patient has had a tracheostomy, **0** otherwise) + |
| **0.72893544** * (**1** if patient has a central line in place, **0** otherwise) + |
| **0.66294066** * (**1** if patient has a chest tube in place, **0** otherwise) + |
| **0.55791264** * (**1** if patient has an ostomy in place, **0** otherwise) + |
| **0.87298112** * (**1** if patient is on ECMO, **0** otherwise) |
